# Construction of epilepsy diagnosis model based on cell senescence-related genes and its potential mechanism

**DOI:** 10.3389/fneur.2025.1555586

**Published:** 2025-05-30

**Authors:** Xiangyao Gong, Wei Lu, Qihua Xiao, Xiaopeng Wang, Chenchen Cui, Hai Tang

**Affiliations:** ^1^Department of Neurology, The Affiliated Hospital of Xuzhou Medical University, Xuzhou, China; ^2^Epilepsy Center, The Affiliated Hospital of Xuzhou Medical University, Xuzhou, China; ^3^Department of General Practice, The Affiliated Suqian Hospital of Xuzhou Medical University, Suqian, China; ^4^Department of Neurosurgery, The Affiliated Hospital of Xuzhou Medical University, Xuzhou, China

**Keywords:** bioinformatics analysis, cell senescence, diagnosis model, epilepsy, senescence-associated secretory phenotype

## Abstract

**Introduction:**

*Epilepsy* is a chronic brain disease with a certain degree of neurodegeneration and is caused by abnormal discharges of neurons. The mechanism of cell senescence has garnered increasing attention in neurodegenerative diseases. However, the role of cell senescence in the onset and progression of epilepsy is unclear. Therefore, this study constructed a diagnostic model of epilepsy based on cellular senescence-related genes (CSRGs) to analyze their role in disease pathogenesis.

**Methods:**

The differentially expressed genes (DEGs) were screened from the epileptic sample dataset of the gene expression omnibus (GEO) database, and the cellular senescence-related DEGs (CSRDEGs) related to epilepsy were identified by CSRGs crossover. The functional enrichment characteristics of CSRDEGs were analyzed using gene ontology (GO) and Kyoto encyclopedia of genes and genomes (KEGG) enrichment analyses. The differences in biological processes between high and low-risk groups were analyzed using gene set enrichment analysis (GSEA). For model construction, logistic regression, random forest, and least absolute shrinkage and selection operator (LASSO) regression were employed to identify key genes, including ribosomal protein S6 kinase alpha-3 (RPS6KA3), cathepsin D (CTSD), and zinc finger protein 101 (ZNF101). Subsequently, a multifactor logistic regression model was developed to evaluate the risk of epilepsy based on these screened genes.

**Results:**

The model exhibited higher area under the curve (AUC) values in the GSE data sets 143272 and 32534, producing encouraging results. Finally, mRNA-miRNA and mRNA-transcription factors (TFs) networks revealed the potential regulatory mechanism of the selected critical genes in the disease.

**Discussion:**

This study elucidated the possible process of cell senescence in epileptic diseases through bioinformatics analysis, offering a potential target for personalized diagnosis and precise treatment.

## 1 Introduction

*Epilepsy* is a common neurological disorder with a high prevalence worldwide. According to the World Health Organization, there are approximately 50 million individuals with epilepsy globally, and millions of new cases emerge annually. The incidence of epilepsy varies significantly among different regions and populations. Generally, epilepsy is more common in developing and low-income countries than in developed ones. This may be due to various factors, including a lack of medical resources, limited diagnosis, and insufficient disease management. Furthermore, epilepsy has a relatively higher incidence rate among children and the elderly. It is one of the primary causes of neurological disorders in children, significantly affecting the quality of life and social functioning of patients (1). Epilepsy has a high incidence rate currently, but the treatment effect is unsatisfactory (2). Therefore, it is crucial to investigate its molecular mechanisms further to identify novel therapeutic targets.

Cell senescence is regarded as one of the key pathological mechanisms in neurodegenerative diseases (3, 4), such as Alzheimer’s disease (AD) (5–7) and Parkinson’s disease (PD) (3, 8). Neurons and glial cells in AD and PD often display typical aging characteristics, including cell cycle arrest, organelle dysfunction, and a pro-inflammatory phenotype (9). Although the role of cellular senescence in neurodegenerative diseases has been extensively studied, its relevance in the pathogenesis of epilepsy has not been fully explored. Some studies in recent years have initially revealed clues indicating that cell senescence may play an important role in epilepsy. Some studies revealed that there are senescent phenotype characteristics (such as upregulation of p16^INK4a^ expression and abnormal activation of cell cycle genes) (10); this implies that cell senescence may be crucial for developing epileptogenic zones and the regulation of seizure frequency. Additionally, the association between epilepsy and neuroinflammation has been widely reported, and the senescence-associated secretory phenotype (SASP) cellular may be a major contributor in promoting the formation of a chronic inflammatory environment (11). As a result, investigating the role of cell senescence-related genes (CSRGs) in epilepsy may assist in identifying specific mechanisms behind the onset and progression of epilepsy and providing a theoretical basis for exploring new therapeutic targets.

In light of this, this study aimed to systematically analyze the functions of CSRGs and explore their potential role in the pathogenesis of epilepsy. Considering the critical role of CSRGs in other neurological diseases, we hypothesized that they may also be crucial in the pathogenesis of epilepsy. This study will use bioinformatics analysis to identify differentially expressed CSRGs related to epilepsy and investigate their regulatory mechanisms in neuronal function and inflammatory response. This study may offer a theoretical foundation for further understanding the molecular mechanisms underlying epilepsy and exploring new therapeutic targets.

## 2 Materials and methods

### 2.1 Data collection and preprocessing

This study acquired three epilepsy-related *Homo sapiens* datasets from the Gene Expression Omnibus (GEO) database^1^ and Gene Set Enrichment (GSE) GSE143272, GSE32534, and GSE4290, using the R package GEOquery (12). The chip platform of the GSE143272 dataset was GPL10558, and the chip platform of datasets GSE32534 and GSE4290 was GPL570. The specific details are given in Table 1. Whole blood tissue served as the tissue source for dataset GSE143272, including 34 untreated epilepsy samples and 51 healthy control samples. Peritumoral cortical tissue was the source of dataset GSE32534, which contains five epilepsy samples and five control samples. The dataset GSE4290 originated from brain tissue containing 23 epilepsy samples.

**Table 1:** GEO microarray chip information.

Data set	GSE143272	GSE32534	GSE4290
Platform	GPL10558	GPL570	GPL570
Species	*Homo sapiens*	*Homo sapiens*	*Homo sapiens*
Tissue	Whole blood tissues	Peritumoral cortex tissues	Brain tissues
Samples in epilepsy group	34	5	23
Samples in control group	51	5	/
Reference	PMID: 30826443 PMID: 32054883	PMID: 23418513	PMID: 16616334

This study aimed to identify genes associated with cellular senescence by collecting protein-coding CSRGs from the GeneCards database.^2^ Protein-coding genes were screened and retained by using “cell senescence” as the search keyword, identifying 3,575 CSRGs. Additionally, relevant literature was searched in PubMed^3^ using the same keyword, and 279 genes related to the cellular aging process in different studies were further collected. After merging and deduplication, 3,619 CSRGs were obtained. The detailed information is depicted in Supplementary Table 1.

Datasets GSE143272 and GSE32534 were preprocessed for standardized and normalized probe annotations using the R package limma (13). A subset of samples from dataset GSE143272 and all samples from dataset GSE32534 were included in this analysis. Furthermore, epilepsy-related samples in dataset GSE4290 served as a validation set for subsequent analyses.

### 2.2 Differentially expressed genes analysis

The samples were divided into two groups based on their grouping in the GSE143272 and GSE32534 datasets: an epilepsy group and a control group. Differential expression between epilepsy and control groups was analyzed using the R package limma. The threshold for identifying differentially expressed genes (DEGs) was established at |logFC| >0.10 and *p*-value <0.05. Specifically, genes were categorized as upregulated if their logFC >0.10 and *p*-value <0.05 and as downregulated if their |logFC| <−0.10 and *p*-value <0.05. The results of differential expression analysis were displayed using volcano plots generated by the R package ggplot2 (14).

Variance analysis was first used to identify DEGs from the GSE143272 and GSE32534 datasets in order to identify cellular senescence-related DEGs (CSRDEGs) associated with epilepsy. The screening criteria included |logFC| >0.10 and *p*-value <0.05. Subsequently, these DEGs were analyzed for intersection with the CSRGs of all epilepsy samples in the GSE4290 dataset. CSRDEGs were finally identified by constructing a Venn diagram displaying the overlap of these genes. Heatmaps were created using the R package pheatmap (15) to display these CSRDEGs.

### 2.3 Gene Ontology and Kyoto Encyclopedia of Genes and Genomes enrichment analysis

Gene Ontology (GO) analysis is a commonly used method for large-scale functional enrichment studies, including cellular component (CC), biological process (BP), and molecular function (MF). The Kyoto Encyclopedia of Genes and Genomes (KEGG) is currently the most widely used database for storing information on genomes, biological pathways, diseases, and drugs. Enrichment analysis of CSRDEGs was performed using GO and KEGG pathway analysis using the R package clusterProfiler (16). The Screening criteria for statistical significance were defined as *p*-value <0.05 and false discovery rate (FDR) (*q*-value) <0.25.

### 2.4 Gene set enrichment analysis

Gene set enrichment analysis (GSEA) is used to evaluate the distribution trends of genes in predefined gene sets ranked based on their correlation with a specific phenotype to determine their contribution to a given phenotype. In this study, the genes in the GSE143272 dataset were first arranged according to their logFC values. Subsequently, GSEA analysis was conducted on all genes in the GSE143272 dataset using the R package clusterProfiler. The parameters of the GSEA analysis were set as follows: the seed value was 2,020, the number of calculations was 1,000, the minimum number of genes contained in each gene set was 10, and the maximum number of genes was 500. The c2 gene set (Cp. All. V2022.1. Hs. Symbols) was obtained through the Molecular Signatures Database. GSEA used gene matrix transposed (GMT) files containing all canonical pathways (3,050 pathways). The screening criteria for significant enrichment were set as adjusted *p*-value <0.05 and FDR value (*q*-value) <0.25, with *p*-value correction using the Benjamini–Hochberg (BH) method (17).

Epilepsy samples in the GSE4290 dataset were divided into high- and low-risk groups based on the median logistic risk score. Differential expression analysis was performed using the R package limma, and genes with |logFC| >0.10 and *p*-value <0.05 in high- and low-risk groups were eliminated. This research focuses on developing a diagnostic support model for epilepsy that differentiates between patients with epilepsy and healthy individuals based on gene expression profiles linked to cellular aging. The model produces scores designed to assess the probability of epilepsy during diagnosis rather than evaluate disease progression or recurrence risk.

### 2.5 Screening key genes

An initial single-factor logistic regression analysis was performed on CSRDEGs, utilizing a screening criterion of *p*-value <0.05 to identify key genes and construct a diagnostic model. The CSRDEGs identified through this screening were subsequently analyzed using a random forest (RF) approach. RF (18), an ensemble learning algorithm, integrates multiple decision trees through bootstrap aggregation and is frequently employed in model construction to generate numerous decision trees, with the final prediction determined by majority voting. The expression levels of CSRDEGs in the GSE143272 dataset were filtered using single-factor logistic regression, and the model was developed using the RF package with the parameters “set.seed (234)” and “ntree = 500.” The “MeanDecreaseGini (19)” metric, which assesses the average reduction in node impurity for a variable across all decision trees, was employed to evaluate the importance of each variable within the model. A higher MeanDecreaseGini value indicates greater variable importance. To determine the optimal number of variables, five 10-fold cross-validations were conducted, and the resulting cross-validation curves were combined. Cross-validation is a method of evaluating model performance by dividing the data into different training and validation sets multiple times, helping to alleviate the problems of overfitting and insufficient training data. Cross-validation was performed using the training set, and the number of variables was selected that minimized the error. The significant variables were selected for subsequent analysis based on the MeanDecreaseGini value.

The least absolute shrinkage and selection operator (LASSO) regression analysis (20) was carried out using the R package glmnet (21). The parameters were adjusted to “set.seed (500)” and running the number of times to 200 to avoid overfitting. LASSO regression helps reduce overfitting and enhances the generalizability of the model by adding a penalty term (lambda times the absolute coefficient value) to the linear regression model. The results of the LASSO regression analysis are visualized through diagnostic model plots and variable trajectory plots. CSRDEGs included in the final LASSO regression model were considered key genes for subsequent analyses. The regulatory mechanisms of key genes were thoroughly explored through these network analyses, and their potential functions in epilepsy pathology were revealed.

### 2.6 Constructing mRNA-miRNA and mRNA-transcription factors (TFs) interaction network

The regulatory mechanisms of key genes and their potential roles in epilepsy pathology were explored by constructing mRNA-miRNA and mRNA-TF interaction networks to identify the upstream regulators and downstream regulatory targets of these key genes. The miRWalk database was used^4^ to predict potential miRNA regulatory molecules of key genes. A total of 72 miRNAs related to 11 key genes were screened out, and the mRNA-miRNA interaction network was constructed and visualized by Cytoscape software. The results demonstrated that ribosomal protein S6 kinase alpha-3 (RPS6KA3) has a significant regulatory association with multiple miRNAs, such as hsa-miR-30c-5p and hsa-miR-19a-3p, indicating that these miRNAs may participate in the pathogenesis of epilepsy by regulating the expression of key genes. Additionally, the interactions of key genes with their transcription factors (TFs) were predicted using the ChIPBase database (22).^5^ The 40 TFs that were identified encompassed JUN, FOS, and STAT3, among others, which interact with 13 key genes. Cytoscape software was used to construct an mRNA-TF interaction network, exhibiting the regulatory network association between RPS6KA3, zinc finger protein 101 (ZNF101), IL7R, and other genes and these TFs. This result suggested that these TFs may play an important role in the pathological process of epilepsy by regulating the expression of key genes.

### 2.7 Key genes for building diagnostic logistic regression models

Logistic regression models are often used to analyze the relationship between independent variables and binary dependent variables. In this study, all key genes were included to construct a multifactor logistic regression model. The coefficient of each key gene in the model was multiplied by its corresponding expression level, and the results were added to calculate the risk score of each sample. The dataset was divided into high- and low-risk groups based on the median risk score. The risk score is calculated as follows:

$$\text{Risk score}=\sum_{i}\text{Coefficient}\;({\text{gene}}_{i})*\text{mRNA expression}\;({\text{gene}}_{i})$$

Nomogram (23) is a graphical tool that uses a set of non-overlapping line segments to represent the functional relationship between multiple independent variables in a rectangular coordinate system. A nomogram was plotted based on the results of the multivariate logistic regression model using the R package rms (24) to demonstrate the interrelationships between key genes included in the model.

Decision curve analysis (DCA) is a method used to evaluate the clinical utility of predictive models, diagnostic tests, and molecular markers. DCA was performed on key genes of the GSE143272 dataset using the R package ggDCA, and decision curve plots were constructed.

Furthermore, ROC curves of the logistic risk scores in GSE143272 and GSE32534 datasets were plotted using the R package pROC (25), and the area under the curve (AUC) values were calculated. The ROC curve is a tool used to evaluate model performance by analyzing the trade-off between sensitivity and specificity to select the optimal model, eliminate suboptimal models, or determine the optimal threshold within a single model. The ROC curve comprehensively measures the ability of the test to distinguish different conditions. The AUC can be used to evaluate the diagnostic performance of the logistic risk score for epilepsy. It is usually between 0.5 and 1. The diagnostic performance improves when the AUC is closer to 1. Low accuracy is indicated by an AUC between 0.5 and 0.7, moderate accuracy by an AUC between 0.7 and 0.9, and high accuracy by an AUC above 0.9.

Semantic comparison of GO annotations provides a quantitative method for assessing similarities between genes and genomes, which has become an essential basis for many bioinformatics analysis methods. This study used the R package GOSemSim (26) to calculate the functional correlations (Friends) of key genes and determine their relationships through functional similarity analysis.

### 2.8 Key gene differential expression verification

The differences in key gene expression between epilepsy and control groups in the GSE143272 and GSE32534 datasets were analyzed using the Mann–Whitney *U* test (27) (Wilcoxon rank sum test). The differential analysis results were visualized using comparison plots between groups, plotted by the R package ggplot2. R package pROC was used to generate the ROC curves of key genes between epilepsy and control groups in GSE143272 and GSE32534 datasets. The AUC values were calculated to evaluate the diagnostic effect of key gene expression in patients with epilepsy.

### 2.9 Statistical analyses

The data processing and analyses in this study were performed using R software (version 4.2.2). Unless otherwise indicated, the independent samples *t*-test (Student’s *t*-test) was used to determine statistical significance for comparisons of continuous variables between two groups if the variables were normally distributed. The Mann–Whitney *U* test (Wilcoxon rank sum test) was used to analyze variables that did not follow the normal distribution. Kruskal–Wallis test was used when comparing three or more groups (28). Correlation coefficients between different molecules were calculated using Spearman correlation analysis (29). Unless otherwise stated, all statistical *p*-values are two-sided, and *p*-values <0.05 were considered statistically significant.

## 3 Results

### 3.1 Processing of epilepsy datasets

**Figure 1:**
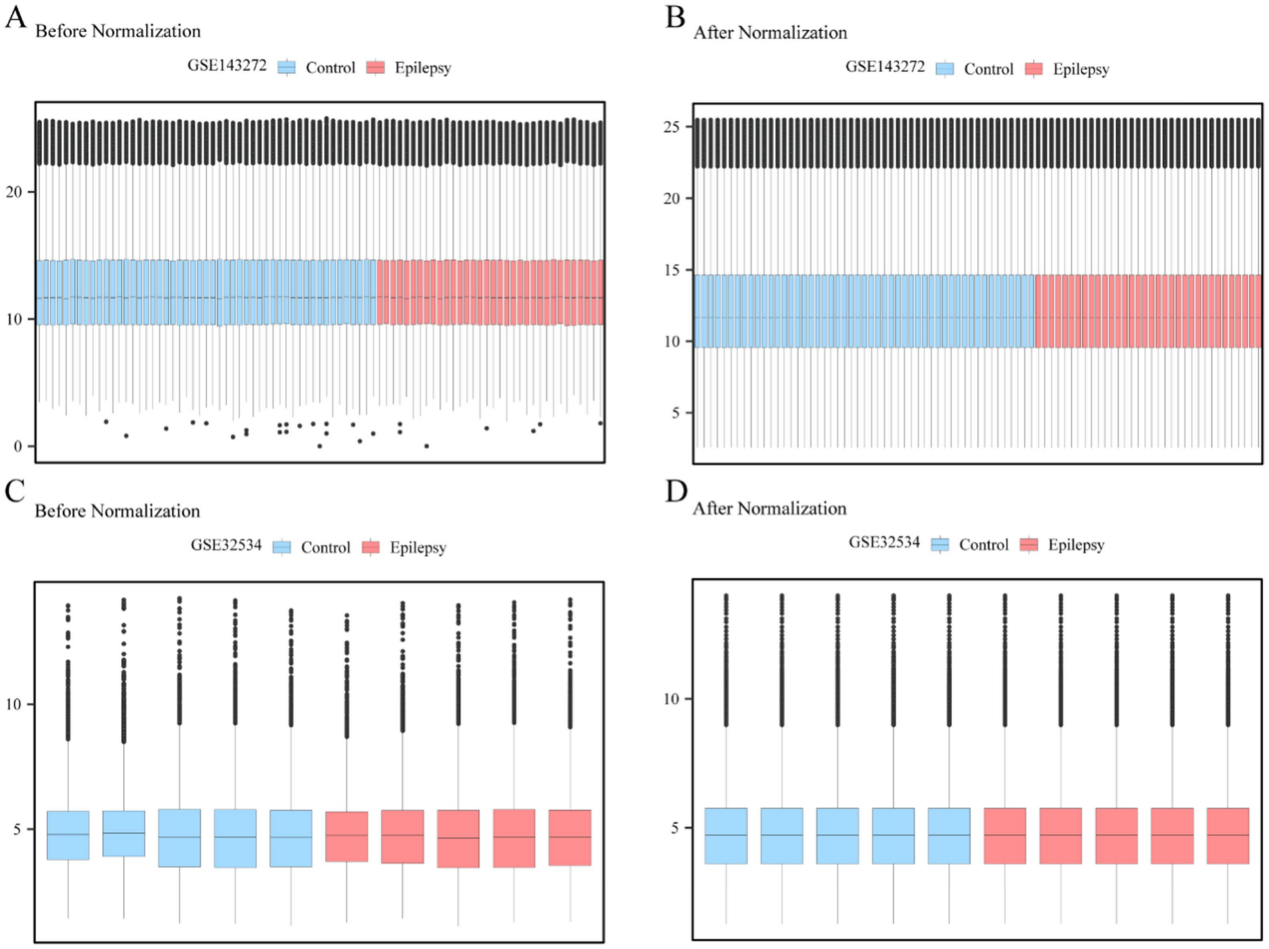
Data to batch processing. **(A,B)** Boxplot of GSE143272 distribution of epilepsy dataset before **(A)** and after **(B)** going to batch. **(C,D)** Epilepsy dataset GSE32534 distribution boxplot before **(C)** and after **(D)** debatched. Light blue is the control group, and light red is the epilepsy group.

First, the batch effect was removed from the epilepsy datasets GSE143272 and GSE32534 using the R package sva (30). The effectiveness of this process was evaluated by comparing expression levels before and after batch effects were eliminated via distribution box plots. Results for dataset GSE143272 are displayed in Figures 1A,B, whereas the results for dataset GSE32534 are displayed in Figures 1C,D. Boxplot analysis revealed that the batch effect of the epilepsy dataset had been effectively eliminated; therefore, the distribution boxplot confirmed the accuracy and reliability of gene expression data analysis.

### 3.2 DEGs related to epileptic cell senescence

**Figure 2:**
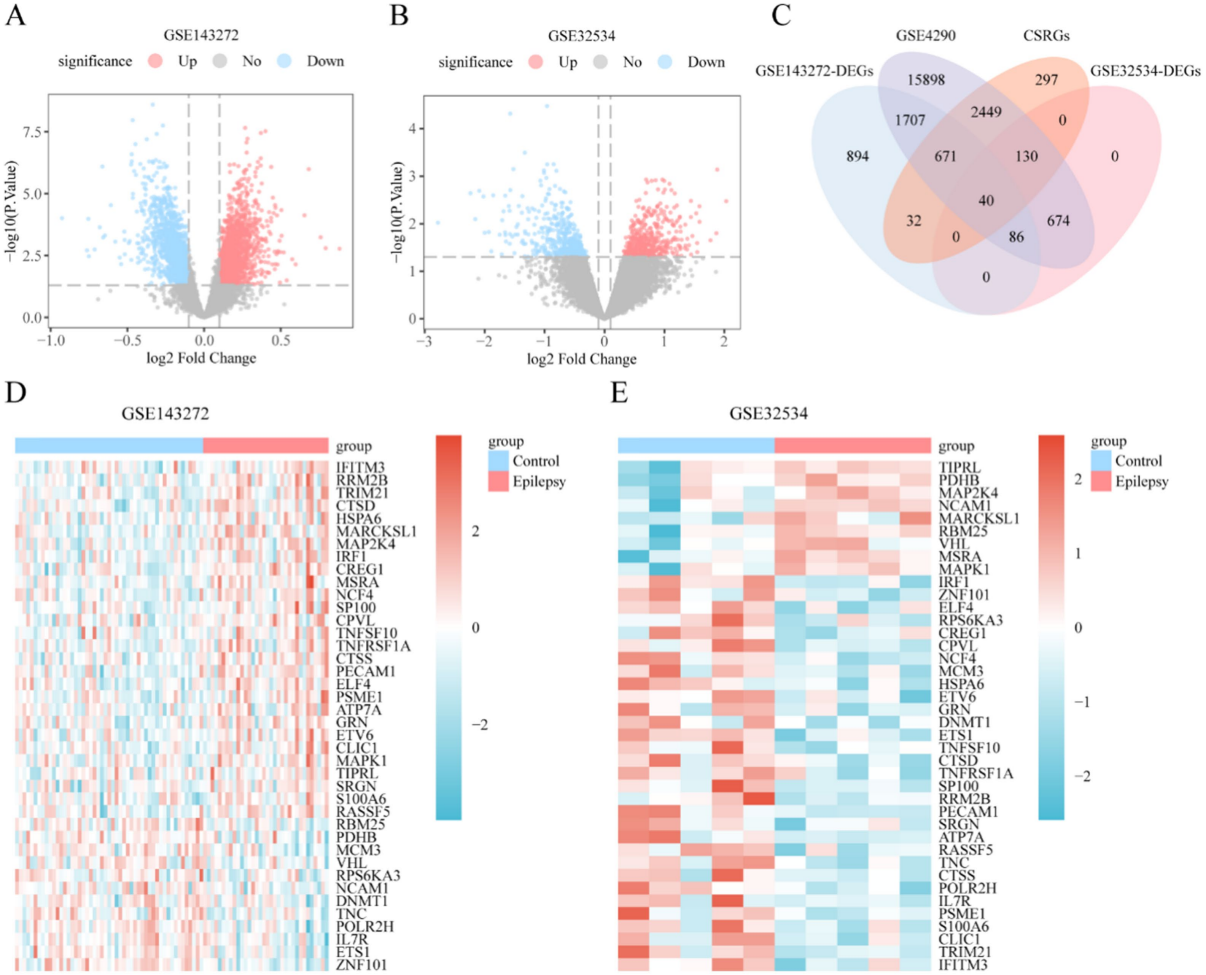
Differential gene expression analysis. **(A,B)** Volcano plot of differentially expressed genes analysis between epilepsy group and control group in GSE143272 **(A)** and GSE32534 dataset **(B)**. **(C)** DEGs in GSE143272 and GSE32534 datasets, genes and CSRGs Venn diagram of all epilepsy samples in GSE4290 dataset. **(D,E)** Heat map of CSRDEGs in GSE143272 **(D)** and GSE32534 datasets **(E)**. DEGs, differentially expressed genes; CSRGs, cellular senescence-related genes; CSRDEGs, cellular senescence-related differentially expressed genes. Light red is the epilepsy group; light blue is the control group. In the heat map, red represents high expression, and blue represents low expression.

Differentiated expression analysis was performed to analyze gene expression differences in GSE143272 and GSE32534 datasets between epilepsy and control groups using the R package limma. A total of 3,430 DEGs were identified in the GSE143272 dataset. The filtering criteria included |logFC| >0.10 and *p*-value <0.05, including 1,631 upregulated (logFC >0.10 and *p*-value <0.05) and 1,799 downregulated genes (logFC <−0.10 and *p*-value <0.05). The results are displayed by a volcano plot (Figure 2A). Moreover, 930 DEGs were identified in the GSE32534 dataset with the same screening criteria, which included 458 upregulated and 472 downregulated genes, and the results are displayed in the volcano plot (Figure 2B).

The intersection analysis of the DEGs of GSE143272 and GSE32534 and the CSRGs in the GSE4290 dataset was conducted to draw a Venn diagram (Figure 2C) to identify CSRDEGs related to cellular senescence. Ultimately, 40 CSRDEGs were identified, including ZNF101, cathepsin D (CTSD), ribonucleotide reductase subunit M2 (RRM2B), anti-RNA polymerase II subunit H (POLR2H), myristoylated alanine-rich C kinase substrate like 1 (MARCKSL1), ETS proto-oncogene 1 (ETS1), cellular repressor of E1A-stimulated genes 1 (CREG1), tripartite motif-containing protein 21 (TRIM21), mitogen-activated protein kinase kinase 4 (MAP2K4), platelet endothelial cell adhesion molecule 1 (PECAM1), methionine sulfoxide reductase A (MSRA), proteasome activator subunit 1 (PSME1), interferon regulatory factor-1 (IRF1), E74-like factor 4 (ELF4), minichromosome maintenance 3 (MCM3), cathepsin S (CTSS), tumor necrosis factor α receptor 1 (TNFRSF1A), chloride intracellular channel protein 1 (CLIC1), E-twenty six variant gene 6 (ETV6), RNA-binding motif protein 25 (RBM25), neutrophil cytosolic factor 4 (NCF4), heat shock 70-kDa protein 6 (HSPA6), speckled protein 100 kDa (SP100), RPS6KA3, ras association domain-containing protein 5 (RASSF5), tenascin-C (TNC), mitogen-activated protein kinase 1 (MAPK1), pyruvate dehydrogenase E1 subunit beta (PDHB), growth factor progranulin (GRN), interleukin-7 receptor (IL7R), ATPase copper-transporting alpha (ATP7A), Von Hippel–Lindau (VHL), tumor necrosis factor superfamily factor 10 (TNFSF10), DNA methyltransferase-1 (DNMT1), interferon-induced transmembrane protein 3 (IFITM3), carboxypeptidase vitellogenic like (CPVL), TOR signaling pathway regulator-like (TIPRL), serglycin (SRGN), neural cell adhesion molecule 1 (NCAM1), and S100 calcium binding protein A6 (S100A6). The expression differences of these CSRDEGs between different sample groups of the GSE143272 and GSE32534 datasets were further analyzed. Heatmaps were drawn using the R package heatmap to present the analysis results (Figures 2D,E).

### 3.3 GO and KEGG enrichment analyses

**Figure 3:**
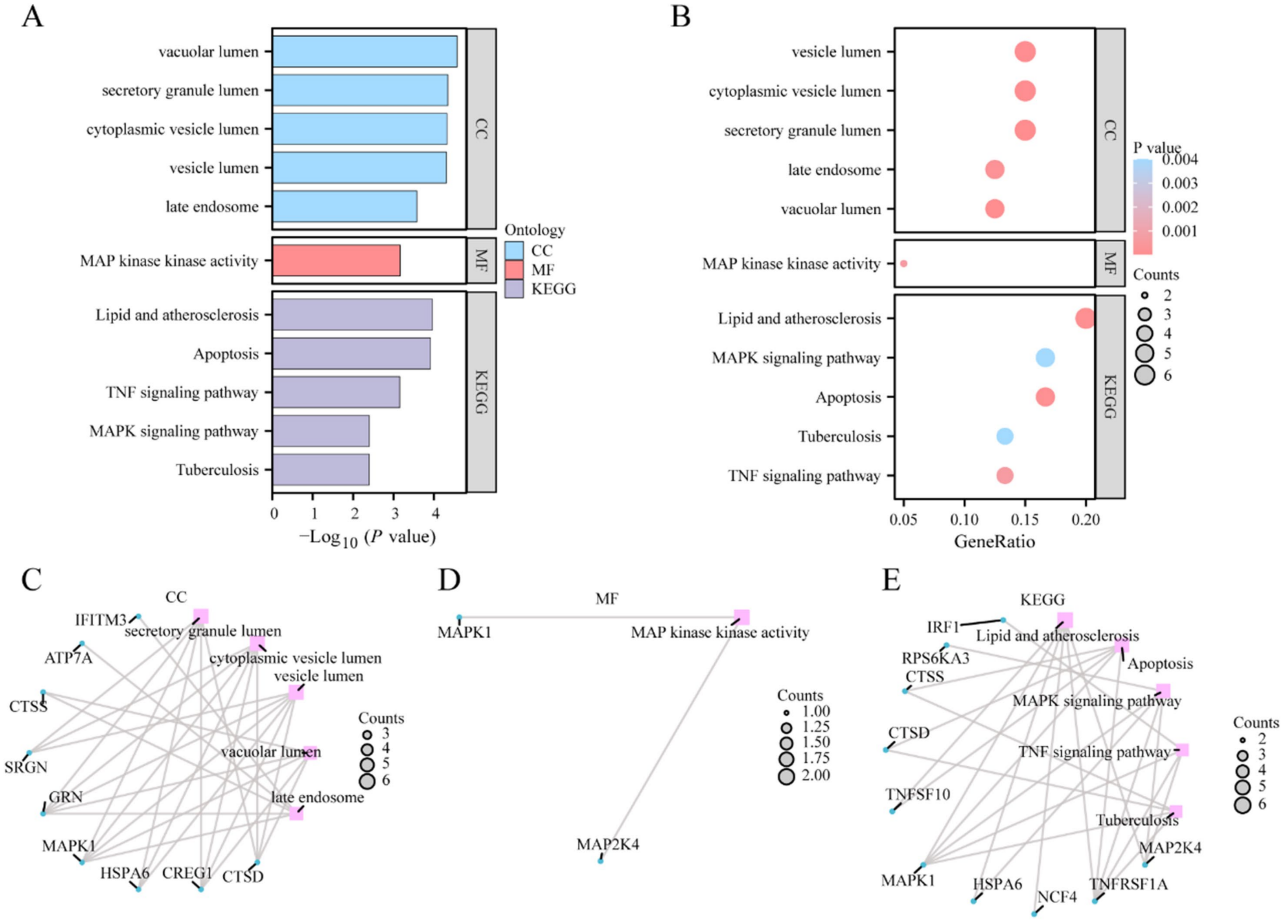
GO and KEGG enrichment analysis of CSRDEGs. **(A,B)** GO and pathway enrichment analysis results of CSRDEGs bar graph **(A)** and bubble plot **(B)** show CC, MF and biological pathway. GO terms and KEGG terms are shown on the ordinate. **(C–E)** GO and pathway (KEGG) enrichment analysis results of CSRDEGs network diagram showing CC **(C)**, MF **(D)**, and KEGG **(E)**. Pink nodes represent items, blue nodes represent molecules, and the lines represent the relationship between items and molecules. CSRDEGs, cellular senescence-related differentially expressed genes; GO, Gene Ontology; KEGG, Kyoto Encyclopedia of Genes and Genomes; CC, cellular component; MF, molecular function. The screening criteria for GO and pathway enrichment analysis were *p*-value <0.05 and FDR value (*q*-value) <0.25.

GO and KEGG enrichment analyses were conducted to explore the association between 40 CSRDEGs and CCs, MFs, and biological pathways associated with epilepsy. The detailed results are presented in Table 2. The analysis indicated that the 40 CSRDEGs were significantly enriched in specific CCs, including the lumen of secretory granules, cytoplasmic vesicles, vacuoles, and late endosomes. Additionally, these genes exhibited enrichment in MFs, notably MAP kinase activity. Crucially, the CSRDEGs were intricately associated with several key biological pathways, as identified by KEGG, including lipid metabolism and atherosclerosis, regulation of apoptosis, the MAPK signaling pathway, and the TNF signaling pathway. The GO and pathway enrichment analysis results were visualized by bar graphs and bubble plots (Figures 3A,B).

**Table 2:** Results of GO and KEGG enrichment analysis for CSRDEGs.

Ontology	ID	Description	Gene ratio	Bg ratio	*p*-value	*p*. adjust	*q*-value
CC	GO:0034774	Secretory granule lumen	6/40	322/19594	4.5061 × 10^−5^	0.00170598	0.0010723
CC	GO:0060205	Cytoplasmic vesicle lumen	6/40	325/19594	4.7447 × 10^−5^	0.00170598	0.0010723
CC	GO:0031983	Vesicle lumen	6/40	327/19594	4.9093 × 10^−5^	0.00170598	0.0010723
CC	GO:0005775	Vacuolar lumen	5/40	174/19594	2.6671 × 10^−5^	0.00170598	0.0010723
CC	GO:0005770	Late endosome	5/40	283/19594	0.00026391	0.00611396	0.00384293
MF	GO:0004708	MAP kinase activity	2/40	18/18410	0.00068893	0.09438274	0.08412138
KEGG	hsa05417	Lipid and atherosclerosis	6/30	215/8164	0.00010881	0.01032613	0.00843993
KEGG	hsa04210	Apoptosis	5/30	136/8164	0.00012148	0.01032613	0.00843993
KEGG	hsa04010	MAPK signaling pathway	5/30	294/8164	0.00397777	0.08501609	0.06948683
KEGG	hsa04668	TNF signaling pathway	4/30	112/8164	0.00069861	0.03958799	0.03235674
KEGG	hsa05152	Tuberculosis	4/30	180/8164	0.00400076	0.08501609	0.06948683

GO, Gene Ontology; CC, cellular component; MF, molecular function; KEGG, Kyoto Encyclopedia of Genes and Genomes; CSRDEGs, cellular senescence-related differentially expressed genes.

Meanwhile, GO enrichment analysis was used to construct the network diagram of CC, MF, and biological pathways (Figures 3C–E). The lines represent the corresponding molecules and the annotations of the corresponding entries, and the larger the nodes, the more molecules the entries contain.

### 3.4 GSEA

**Figure 4:**
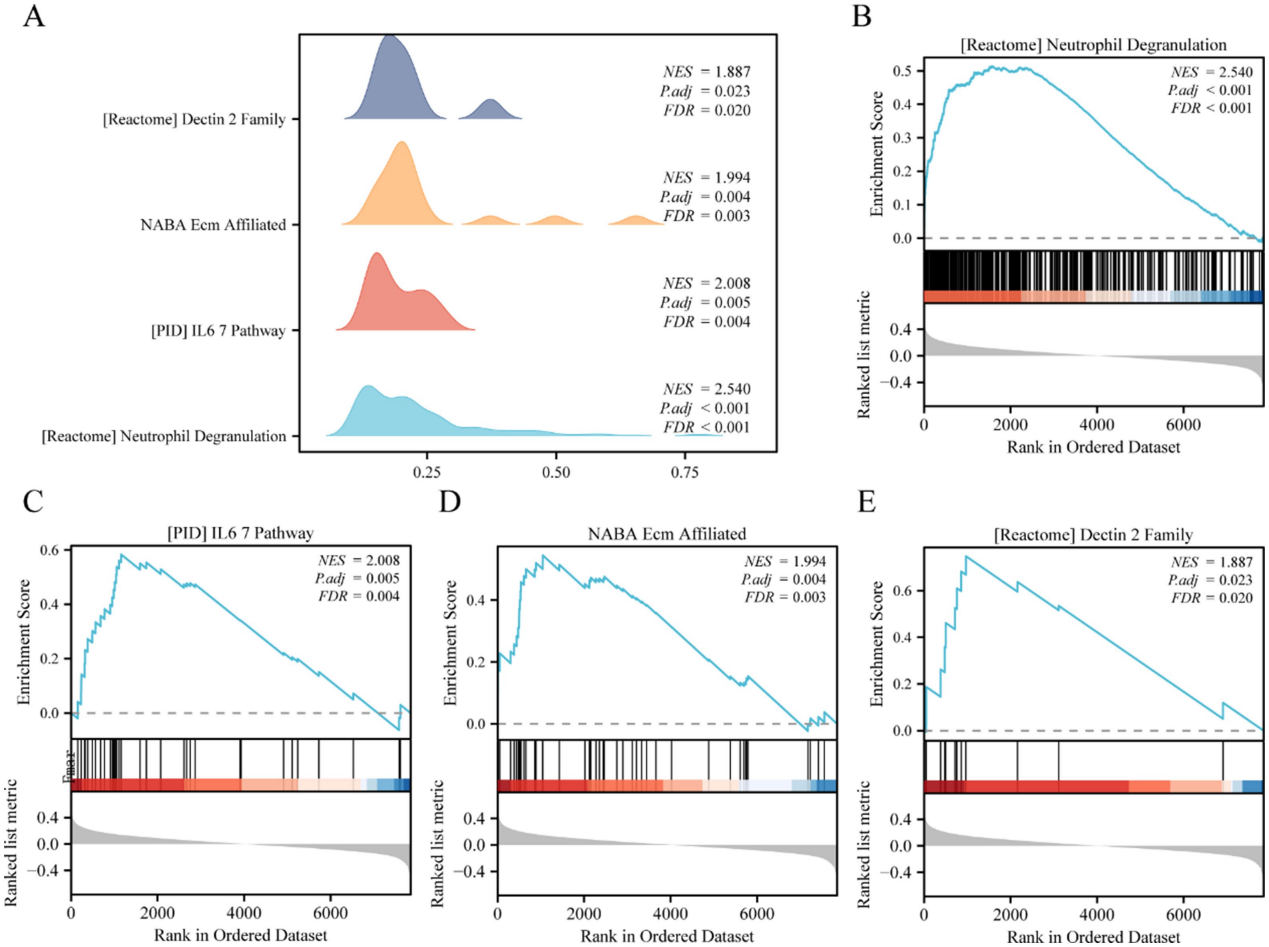
GSEA analysis of epilepsy. **(A)** GSEA mountain plot of 4 biological functions of dataset GSE143272. **(B–E)** Gene set enrichment analysis (GSEA) showed that all genes were significantly enriched in neutrophil degranulation **(B)**, IL6 7 pathway **(C)**, NABA ECM affiliated **(D)**, and Dectin 2 family **(E)**. GSEA, gene set enrichment analysis. The screening criteria of GSEA were adj. *p*-value <0.05 and FDR value (*q*-value) <0.25, and the *p*-value correction method was Benjamini–Hochberg (BH).

GSEA analysis was used to study the association between the expression levels of all genes in the GSE143272 dataset and the BPs, CCs, and MFs in which they were involved (Figure 4A); this helped determine the effects of expression levels of all genes in the GSE143272 dataset on epilepsy. The specific results are displayed in Table 3. The findings revealed that all genes in the GSE143272 dataset were significantly enriched in neutrophil degranulation (Figure 4B), IL6 7 pathway (Figure 4C), and NABA extracellular matrix (ECM) affiliated (Figure 4D). Dectin 2 family (Figure 4E) and other biologically related functions and signaling pathways.

**Table 3:** Results of GSEA for GSE143272.

ID	Set size	Enrichment score	NES	*p-*value	*p*. adjust	*q*-value
REACTOME_NEUTROPHIL_DEGRANULATION	379	0.51414888	2.53977724	12 × 10^−10^	2.1178 × 10^−8^	1.7696 × 10^−8^
PID_IL6_7_PATHWAY	34	0.58337638	2.00811468	0.00011881	0.00503238	0.004205
NABA_ECM_AFFILIATED	46	0.54504668	1.9943069	9.0572 × 10^−5^	0.00411023	0.00343446
REACTOME_DECTIN_2_FAMILY	11	0.7478177	1.88658268	0.00111907	0.0234389	0.01958527

GSEA, gene set enrichment analysis.

### 3.5 Screening key genes

**Figure 5:**
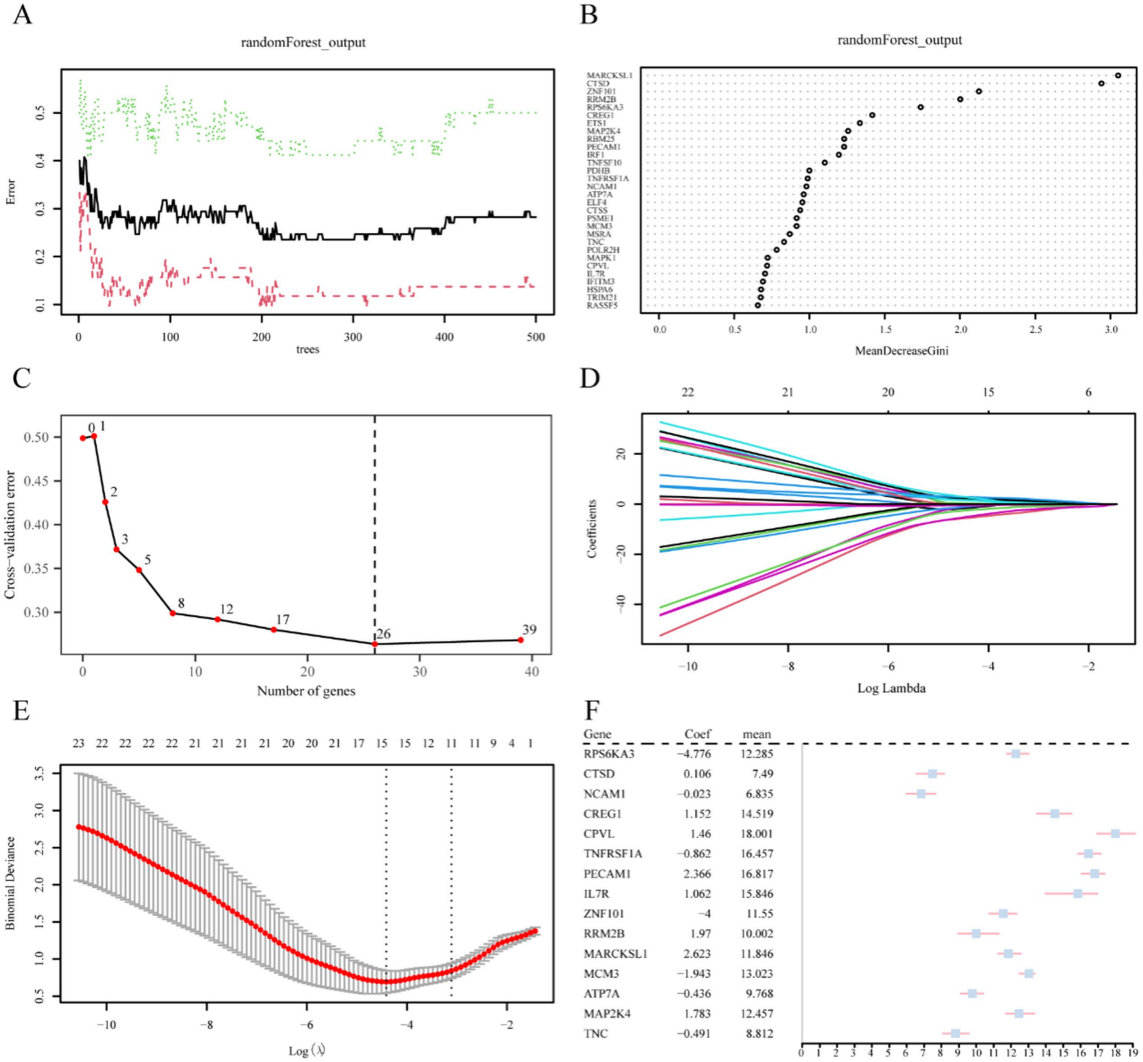
Screening of key genes. **(A)** Plot of model training error of RF algorithm. **(B)** MeanDecreaseGini scatter plot of CSRDEGs (in descending MeanDecreaseGini order). **(C)** Cross-validation error plot. **(D)** Diagnostic model plot of LASSO regression model. **(E)** Variable trajectory plot of LASSO regression model. **(F)** Forest plot of key genes in LASSO regression model. CSRDEGs, cellular senescence-related differentially expressed genes; LASSO, least absolute shrinkage and selection operator.

A multi-step analysis was performed to evaluate the value of 40 CSRDEGs in the diagnosis of reflux. First, single-factor logistic regression was employed to screen 40 CSRDEGs using a *p*-value <0.05 as a screening criterion. The results demonstrated that 39 significant CSRDEGs were identified (Supplementary Table 2). Subsequently, the expression levels of these 39 CSRDEGs in the engineering group were analyzed and led in the GSE143272 dataset using the RF algorithm. The algorithm was configured with a seed value of 234 and 500 decision trees, analogous to the decision tree workpiece spindle (Figure 5A). The results demonstrate that when the number of decision trees is about 300, the artifacts reach a minimum and stabilize.

Moreover, a MeanDecreaseGini scatter plot was created for the 39 CSRDEGs to select essential genes further (Figure 5B). The average value of the variable reducing node impurity across all trees is represented by MeanDecreaseGini. The significance of the gene in distinguishing the epilepsy group from the control group increases with this value, which in turn has a greater impact on the diagnosis of epilepsy. Besides, a five 10-fold cross-validation analysis determined the optimal number of genes, and the cross-validation error curve was plotted (Figure 5C). The curve represented that the model error was at its lowest when there were 26 genes. Combining these results with the MeanDecreaseGini value, 26 CSRDEGs were selected for further analysis. These selected CSRDEGs significantly influence the diagnosis of epilepsy and are ranked in descending order of importance as follows: MARCKSL1, CTSD, ZNF101, RRM2B, RPS6KA3, CREG1, ETS1, MAP2K4, RBM25, PECAM1, IRF1, TNFSF10, PDHB, TNFRSF1A, NCAM1, ATP7A, ELF4, CTSS, PSME1, MCM3, MSRA, TNC, POLR2H, MAPK1, CPVL, and IL7R (Figures 5B,C).

Then, the 26 CSRDEGs identified by the RF algorithm were subjected to LASSO regression analysis to construct a LASSO risk model. The analysis results were displayed through the LASSO regression model plot (Figure 5D) and the LASSO variable trajectory plot (Figure 5E). The LASSO regression model finally identified the following 15 CSRDEGs: RPS6KA3, CTSD, NCAM1, CREG1, CPVL, TNFRSF1A, PECAM1, IL7R, ZNF101, RRM2B, MARCKSL1, MCM3, ATP7A, MAP2K4, and TNC. A forest plot of key genes was drawn using these 15 genes, which were analyzed further (Figure 5F).

### 3.6 Construction of mRNA-miRNA and mRNA-TF interaction network of key genes

**Figure 6:**
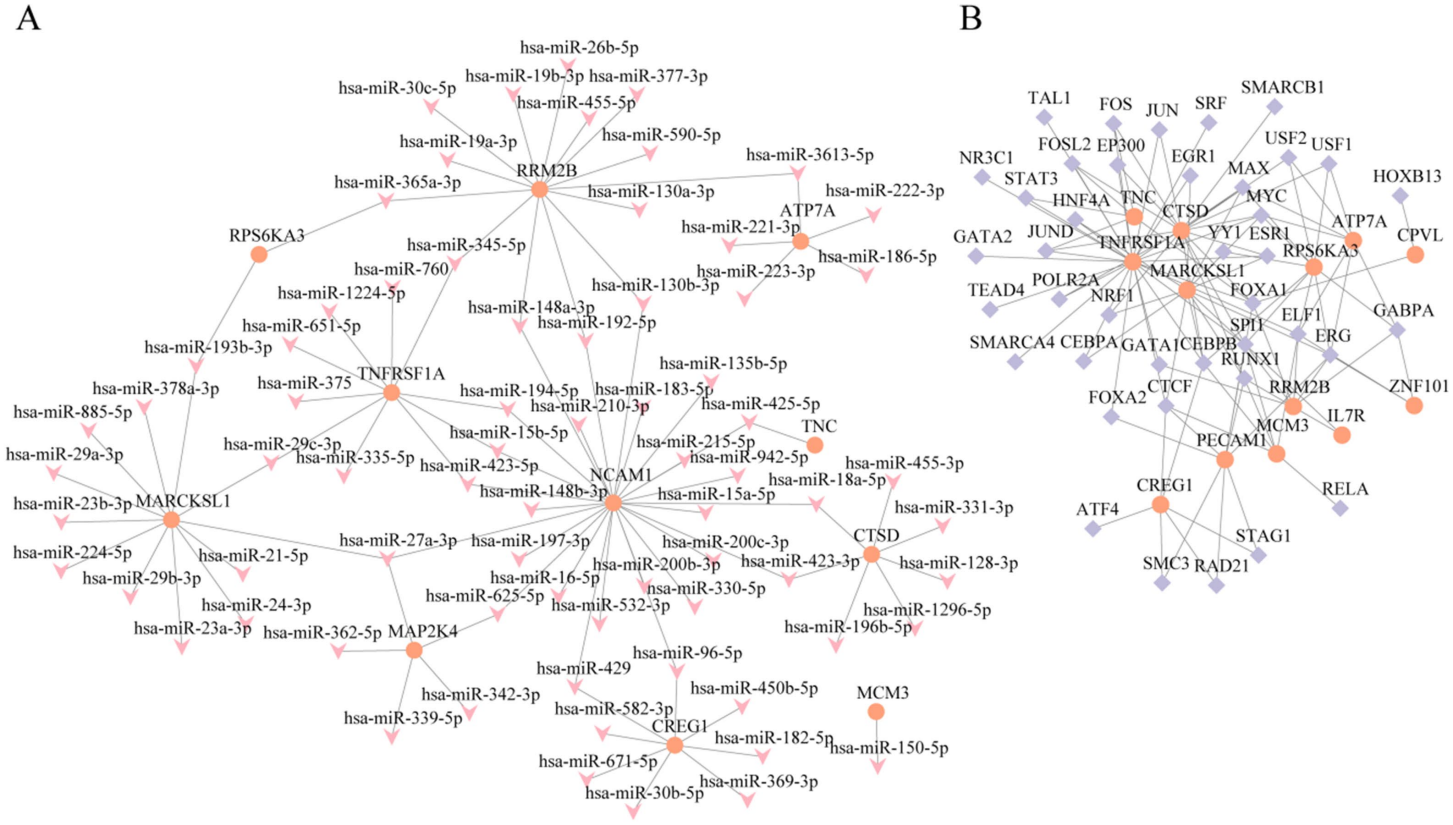
Interaction network analysis of key genes. **(A)** mRNA-miRNA interaction network of key genes. **(B)** mRNA-TF interaction network of key genes. TF, transcription factor. Orange is mRNA, pink is miRNA, and purple is TF.

An interaction network based on mRNA-miRNA and mRNA-TF was designed to further understand the regulatory mechanism of the key genes in epilepsy. Their regulatory patterns and potential molecular mechanisms were thoroughly examined by visualizing them through Cytoscape software. First, the miRNA regulatory associations of the 15 key genes (including RPS6KA3, CTSD, NCAM1, CREG1, CPVL, TNFRSF1A, PECAM1, IL7R, ZNF101, RRM2B, MARCKSL1, MCM3, ATP7A, MAP2K4, and TNC) were predicted using the miRWalk database. Furthermore, an mRNA-miRNA interaction network was built (Figure 6A). The results revealed that 11 key genes (including ATP7A, CREG1, CTSD, MAP2K4, MARCKSL1, MCM3, NCAM1, RPS6KA3, RRM2B, TNC, TNFRSF1A) had significant interactions with 72 miRNAs. Detailed information is provided in Supplementary Table 3.

Then, the TFs binding to these key genes were predicted using the ChIPBase database. An mRNA-TF interaction network was constructed (Figure 6B). The results revealed that 13 key genes (such as RPS6KA3, ATP7A, CPVL, CREG1, CTSD, IL7R, MARCKSL1, MCM3, PECAM1, RRM2B, TNC, TNFRSF1A, and ZNF101) interacted with 40 transcription factors. Further details are documented in Supplementary Table 4. These interaction networks provide new insights into the regulatory mechanisms of these genes and their potential biological functions in epilepsy.

The mRNA-miRNA network is illustrated in Figure 6A. The complex regulatory associations between genes such as RPS6KA3 and MAP2K4 and multiple miRNAs highlight that these miRNAs may play essential roles in the pathological process of epilepsy by regulating gene expression. Figure 6B demonstrates the mRNA-TF network, which exhibited significant interactions between key genes and multiple TFs (such as JUN, FOS, and STAT3, among others), suggesting that these factors may be crucial in the regulatory network of epilepsy by controlling the expression of key genes.

### 3.7 Establishment of diagnostic logistic regression model and functional similarity analysis of key genes

The 15 key genes (RPS6KA3, CTSD, NCAM1, CREG1, CPVL, TNFRSF1A, PECAM1, IL7R, ZNF101, RRM2B, MARCKSL1, MCM3, ATP7A, MAP2K4, and TNC) were incorporated into a multivariate logistic regression model to determine the coefficients of each gene. This analysis facilitated the development of a diagnostic model for epilepsy. Then, the expression and coefficient of 15 key genes from the GSE143272 dataset were interpolated based on the risk score formula. The risk score of each sample was determined, and then the epilepsy group was divided into low- and high-risk scores based on their median risk score value. The following formula was used to calculate the risk score for the GSE143272 dataset:

$$\begin{array}{l} \text{Risk score}=-1549.336*\mathrm{RPS}6\mathrm{KA}3+588.388*\text{CTSD}+\\ 446.008*\text{NCAM}1-94.376*\text{CREG}1+\\ 640.352*\text{CPVL}-722.159*\text{TNFRSF}1A+\\ 17.809*\text{PECAM}1+640.925*\mathrm{IL}7R-\\ 1789.441*\mathrm{ZNF}101+852.298*\mathrm{RRM}2B+\\ 125.355*\text{MARCKSL}1-826.417*\mathrm{MCM}3-\\ 979.354*\mathrm{ATP}7A+1776.780*\mathrm{MAP}2K4+\\ 422.015*\mathrm{TNC} \end{array}$$

**Figure 7:**
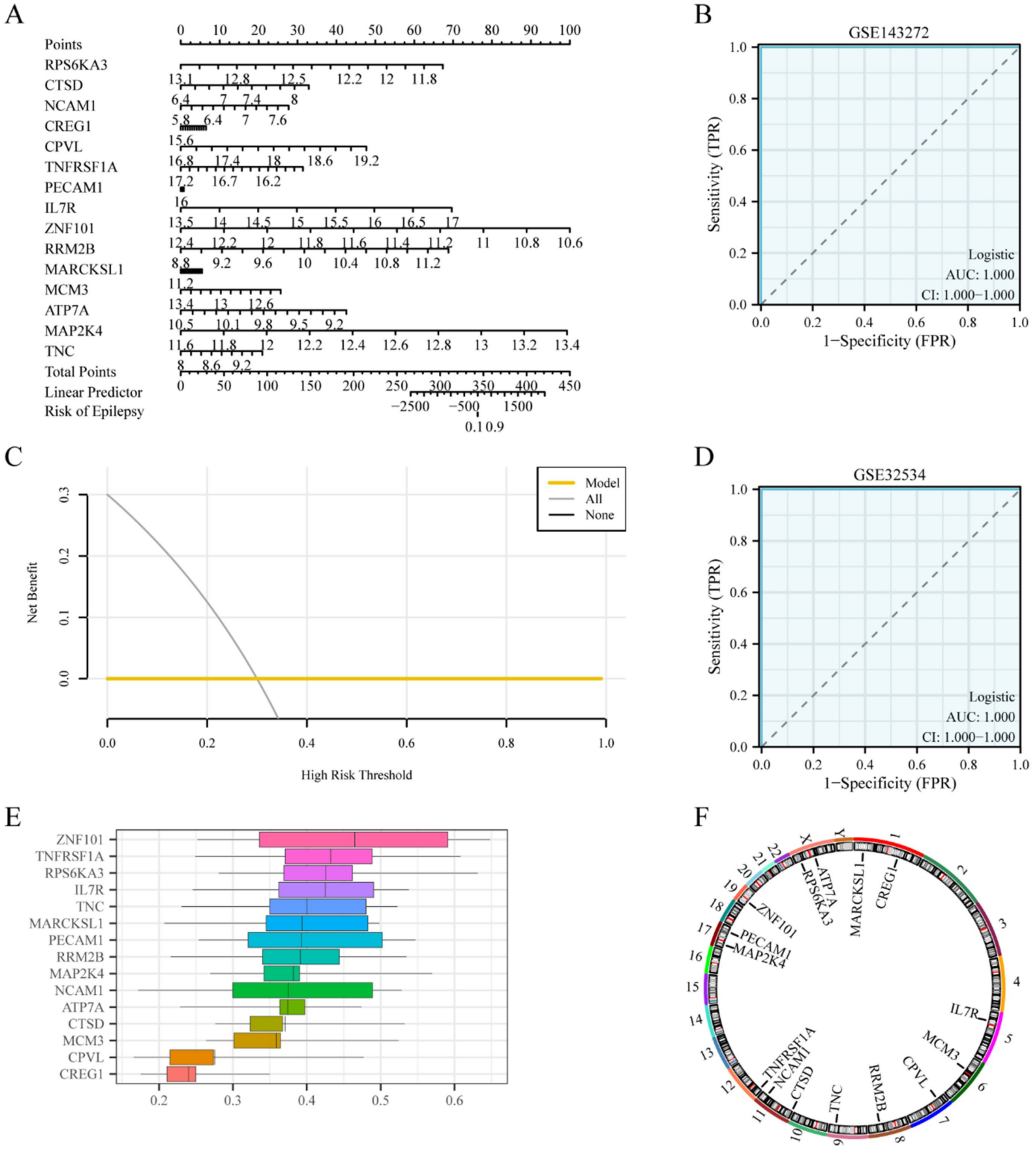
Key genes to construct diagnostic. **(A)** Logistic regression model nomogram of key genes in the diagnostic multivariate logistic model based on dataset GSE143272. **(B)** Diagnostic ROC curve of risk score of diagnostic multivariate logistic model in data set GSE143272. **(C)** DCA plot of the key genes of the diagnostic multivariate logistic model based on dataset GSE143272. **(D)** Diagnostic ROC curve of risk score of diagnostic multivariate logistic model in dataset GSE32534. **(E)** Functional similarity map of key genes. **(F)** Chromosomal mapping of key genes. The ordinate of the DCA plot is the net benefit, and the abscissa is the probability threshold or threshold probability. DCA, decision curve analysis. ROC, receiver operating characteristic; AUC, area under the curve. The closer the AUC is to 1 in the ROC curve, the better the diagnostic performance. When AUC was above 0.9, the accuracy was high.

The association between the 15 key genes was depicted using a nomogram (Figure 7A), which exhibited that ZNF101 and MAP2K4 expression were the main contributors to the multivariate logistic model. Then, the diagnostic performance of the multivariate logistic model for epilepsy was evaluated by DCA based on the GSE143272 dataset. The results are illustrated in Figure 7C, which demonstrates that the line of the model was stable at a higher level than all and none in a certain range, and the net benefit of the model was higher, indicating that the diagnostic effect of the model was better. Finally, the ROC curve was plotted based on the risk scores of the GSE143272 and GSE32534 datasets using the R package pROC to verify the utility of the multifactor logistic model in epilepsy diagnosis. According to ROC analysis, the multifactor logistic model demonstrated high accuracy in epilepsy diagnosis (Figures 7B,D). The functional similarity (Friends) analysis scores were used to determine the genes that play an important role in the biological process of epilepsy (Figure 7E). The results revealed that ZNF101 played an essential role in epilepsy and was the closest to the cut-off value (cut-off value = 0.60).

The location of 15 key genes on the human chromosome was analyzed using the R package RCircos (31), and a chromosome localization map was drawn (Figure 7F), which revealed that more genes were located on chromosomes 1, 11, 17, and X; MARCKSL1 and CREG1 were on chromosome 1, CTSD and NCAM1 on chromosome 11, MAP2K4 and PECAM1 on chromosome 17, and ATP7A and RPS6KA3 on chromosome X.

### 3.8 Differential expression validation analysis of key genes

**Figure 8:**
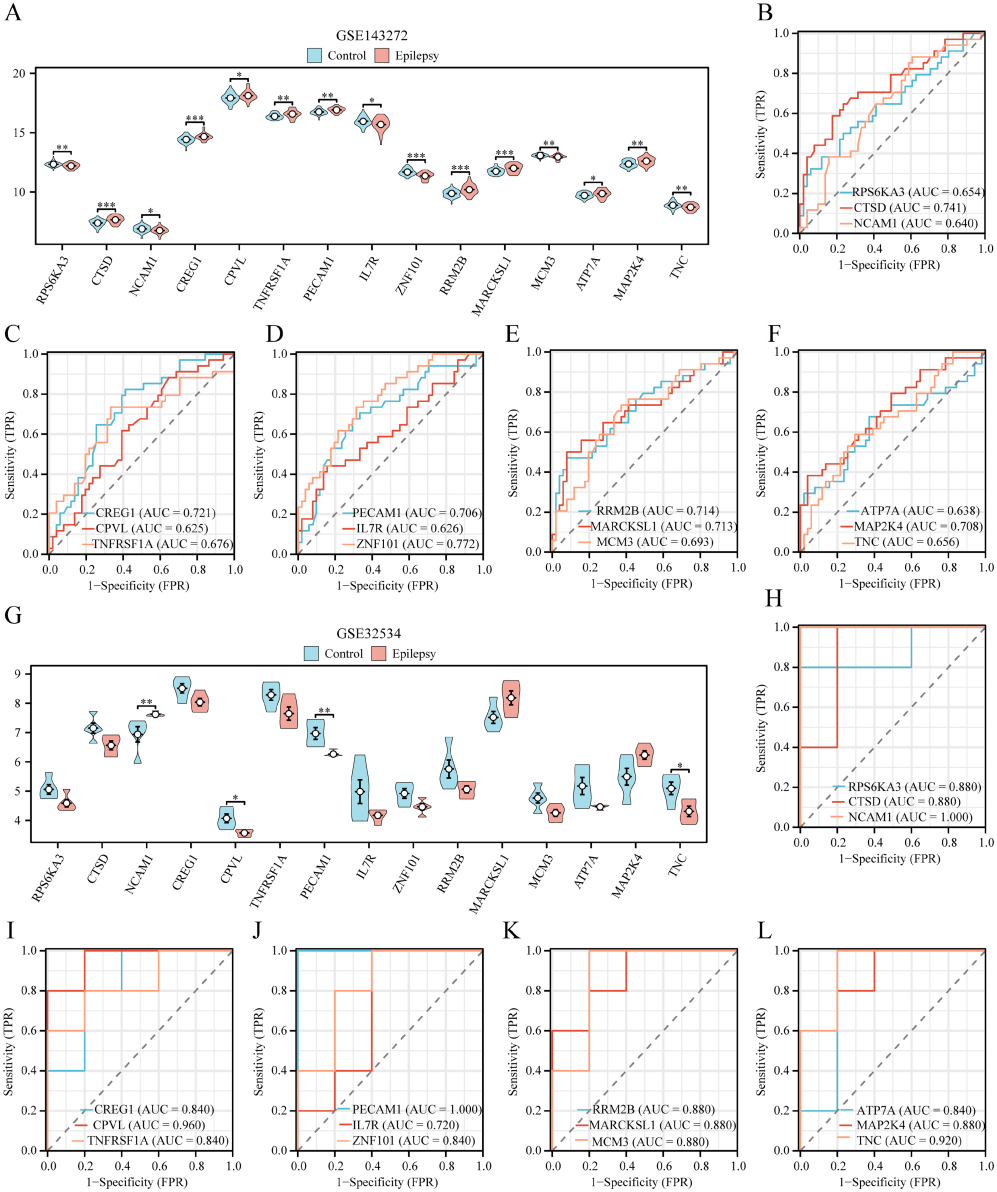
Validation analysis of differential expression of key genes. **(A)** Group comparison of key genes in the epilepsy group and the control group of dataset GSE143272. **(B–F)** Key genes RPS6KA3, CTSD, and NCAM1 **(B)**, CREG1, CPVL, and TNFRSF1A **(C)**, PECAM1, IL7R, and ZNF101 **(D)**, RRM2B, MARCKSL1, and MCM3 **(E)**, ATP7A, ROC curves of MAP2K4 and TNC **(F)** in dataset GSE143272. **(G)** Group comparison diagram of key genes in dataset GSE32534 epilepsy and control groups. **(H–L)** Key genes: RPS6KA3, CTSD, and NCAM1 **(H)**, CREG1, CPVL, and TNFRSF1A **(I)**, PECAM1, IL7R, and ZNF101 **(J)**, RRM2B, MARCKSL1, and MCM3 **(K)**, ATP7A, GSE32534 ROC curves of MAP2K4 and TNC **(L)** in dataset GSE32534. ^*^Represents *p*-value <0.05, indicating statistical significance. ^**^Represents *p*-value <0.01, highly statistically significant. ^***^Represents *p*-value <0.001 and highly statistically significant. When AUC >0.5, it indicates that the molecule’s expression is a trend to promote the event’s occurrence, and the closer the AUC is to 1, the better the diagnostic effect. AUC between 0.5 and 0.7 had low accuracy, AUC between 0.7 and 0.9 had moderate accuracy, and AUC above 0.9 had high accuracy. DCA, decision curve analysis; ROC, receiver operating characteristic; AUC, area under the curve; TPR, true positive rate; FPR, false positive rate. Light blue represents the control group, and light red represents the epilepsy group.

To elucidate the roles of 15 key genes (RPS6KA3, CTSD, NCAM1, CREG1, CPVL, TNFRSF1A, PECAM1, IL7R, ZNF101, RRM2B, MARCKSL1, MCM3, ATP7A, MAP2K4, and TNC) within the GSE143272 dataset, a differential expression analysis was conducted comparing the epilepsy and control cohorts. The findings, depicted in Figure 8A, indicate that the expression levels of five pivotal genes (CTSD, CREG1, ZNF101, RRM2B, and MARCKSL1) demonstrated statistically significant differences (*p* < 0.001) between the epilepsy and control groups. Finally, the R package pROC was used to draw ROC curves based on the expression levels of key genes in the GSE143272 dataset. According to the ROC curves (Figures 8B–F), the expression level of key genes including CTSD, CREG1, PECAM1, ZNF101, RRM2B, MARCKSL1, and MAP2K4 demonstrated a certain accuracy in classifying epilepsy and control groups (0.7 < AUC < 0.9). Moreover, the expression levels of RPS6KA3, NCAM1, CPVL, TNFRSF1A, IL7R, MCM3, ATP7A, and TNC exhibited low accuracy in classifying epilepsy and control groups (0.5 < AUC < 0.7).

By applying the same analytical approach, we examined the expression profiles of key genes (RPS6KA3, CTSD, NCAM1, CREG1, CPVL, TNFRSF1A, PECAM1, IL7R, ZNF101, RRM2B, MARCKSL1, MCM3, ATP7A, MAP2K4, TNC) in the GSE32534 dataset. The differential expression patterns of these 15 key genes between the epilepsy group and the control group were illustrated using a grouped comparison chart (Figure 8G). These findings demonstrated that the expression levels of two key genes, namely NCAM1 and PECAM1, were highly statistically significant (*p*-value <0.01). Finally, the R package pROC was used to draw the ROC curve based on the expression levels of the key genes in the GSE32534 dataset. The ROC curves (Figures 8H–L) revealed that the expression levels of key genes NCAM1, CPVL, PECAM1, and TNC had high accuracy in classifying epilepsy and control groups (AUC >0.9). Furthermore, the expression levels of RPS6KA3, CTSD, CREG1, TNFRSF1A, IL7R, ZNF101, RRM2B, MARCKSL1, MCM3, ATP7A, and MAP2K4 exhibited moderate accuracy (0.7 < AUC < 0.9).

### 3.9 GSEA enrichment analysis based on high and low logistic risk score groups

**Figure 9:**
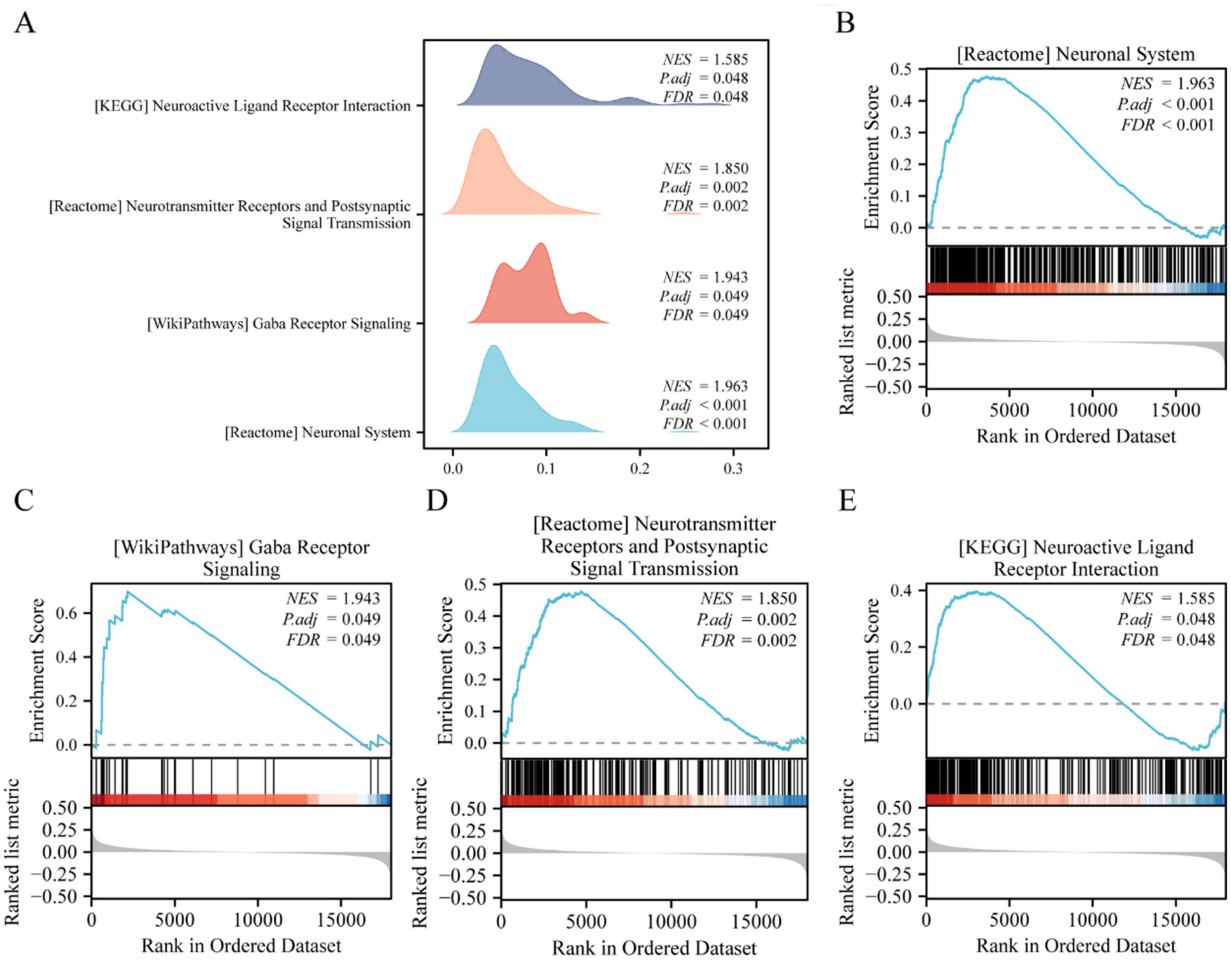
GSEA analysis of the high and low-risk groups of epilepsy. **(A)** GSEA mountain plot of four biological functions of dataset GSE4290. **(B–E)** GSEA showed that all genes were significantly enriched in neuronal system **(B)**, anti inflammatory response GABA receptor signaling **(C)**, and anti-inflammatory response GABA receptor signaling **(C)**. Neurotransmitter receptors and postsynaptic signal transmission **(D)**, neuroactive ligand receptor interaction **(E)**. GSEA, gene set enrichment analysis. The screening criteria of GSEA were adj. *p*-value <0.05 and FDR value (*q*-value) <0.25, and the *p*-value correction method was BH.

GSEA was used to investigate the association between the expression of all 21,655 genes and the BPs, CCs, and MFs involved in different epilepsy risk score groups (low/high) in the GSE4290 dataset (Figure 9A) in order to determine the effect of all gene expression levels in the GSE4290 dataset on the difference between high and low epilepsy risk score groups. The specific results are demonstrated in Table 4. The results revealed that all the genes in the GSE4290 dataset were significantly enriched in the neuronal system (Figure 9B), anti-inflammatory response GABA receptor signaling (Figure 9C), neurotransmitter receptors and postsynaptic signal transmission (Figure 9D), neuroactive ligand-receptor interaction (Figure 9E), and other biologically relevant functions and signaling pathways.

**Table 4:** Results of GSEA (low/high) for GSE4290.

ID	Set size	Enrichment score	NES	*p-*value	*p*. adjust	*q*-value
REACTOME_NEURONAL_SYSTEM	394	0.47660382	1.96345638	1 × 10^−10^	2.479 × 10^−7^	2.4516 × 10^−7^
WP_GABA_RECEPTOR_SIGNALING	30	0.69762082	1.94311111	0.00011952	0.04938266	0.04883643
REACTOME_NEUROTRANSMITTER_RECEPTORS_AND_POSTSYNAPTIC_SIGNAL_TRANSMISSION	196	0.47801477	1.84995884	1.9014 × 10^−6^	0.00157115	0.00155377
KEGG_NEUROACTIVE_LIGAND_RECEPTOR_INTERACTION	262	0.3956592	1.58541008	9.7042 × 10^−5^	0.04811335	0.04758116

GSEA, gene set enrichment analysis.

## 4 Discussion

This study identified 15 key genes linked to epilepsy, such as RPS6KA3 and TNFRSF1A, which are involved in lipid metabolism, apoptosis, and inflammation. These genes may influence seizure development and frequency. Additionally, we explored the role of CSRGs in epilepsy and developed a diagnostic model. Current epilepsy treatments, primarily including drugs and surgery (32), demonstrate varying effectiveness, with about 30% of patients experiencing drug-resistant epilepsy (33). This resistance may result from gene mutations, drug metabolism issues, and molecular changes in the brain (34).

Surgical treatment can control seizures by removing the lesion area, but it requires precise localization of the epileptic focus and has surgical risks, such as possible neurological damage (35). Current epilepsy treatments have limitations, making it essential to explore their molecular mechanisms and identify novel therapeutic targets. The complex pathology of epilepsy involves genetic mutations, neurotransmitter imbalances, and synaptic dysfunction. Studies have identified genes such as SCN1A, GABRA1, and KCNQ2 (36), which regulate neuronal electrophysiological activity, synaptic signaling, and neural development (37). Mutations in the SCN1A gene are a major cause of severe epilepsy in infants (38), while mutations in KCNQ2 and KCNQ3 genes increase neuronal excitability, affecting seizure frequency and type (39). Neuroinflammation and oxidative stress also play crucial roles in epilepsy development (40), with neuroinflammation triggering abnormal neuronal firing and epileptic lesions and oxidative stress causing mitochondrial dysfunction and calcium imbalance (41). Since most studies focused on specific gene mutations or pathways without exploring their interactions, a comprehensive understanding of the etiology and pathogenesis of epilepsy is lacking despite advances in understanding molecular mechanisms. Most studies focus on specific gene mutations or signaling pathways without understanding the interactions between molecular mechanisms. Developing targeted treatments for epilepsy is challenging due to its diverse and individual causes. The global prevalence and treatment difficulties of epilepsy highlight the need for a deeper understanding of its molecular mechanisms for prevention and treatment.

Cellular senescence, an irreversible cell cycle arrest due to stress, was initially seen as protective against tumors but is now linked to various diseases, including cardiovascular, metabolic, and neurodegenerative disorders (42). Recently, research interest has been drawn to CSRGs, which are crucial in aging-related diseases through their effects on the cell cycle, metabolism, and inflammation (43). Studying these genes is vital for understanding complex disease mechanisms. For instance, genes such as p16^INK4a^, p21, and p53 are overexpressed in AD patients, which contribute to the disease through stress response, mitochondrial function, and neuroinflammation (44). Moreover, cellular aging leads to mitochondrial dysfunction and dopamine neuron damage, causing movement and cognitive issues (45). Notably, the SASP, a hallmark of cellular senescence, plays a critical role in establishing a chronic inflammatory microenvironment within the central nervous system. This is mediated by the secretion of various pro-inflammatory cytokines, including tumor necrosis factor-α (TNF-α) and interleukin-6 (IL-6). This inflammatory milieu not only exacerbates glial cell activation and synaptic structural abnormalities but also disrupts GABAergic inhibitory neurotransmission, thereby increasing neuronal synchrony and excitability. These collective alterations contribute to the pathophysiology and recurrence of epilepsy (46–48). Consequently, targeting senescence-related genes and pathways represents a promising therapeutic strategy.

The role of cellular senescence in neurodegenerative diseases such as AD and PD is well-established; its mechanisms in epilepsy remain unclear. Most studies on epilepsy focus on inflammation and neuronal apoptosis (49), rarely addressing cell aging-related genes such as p16^INK4a^, p53, and CDKN2A. The expression patterns of CSRGs in epilepsy patients and their impact on the lesion microenvironment are not well understood. The precise function of cell senescence in different forms of epilepsy, including temporal lobe and infantile epilepsy, also needs further investigation.

This study analyzed gene expression data from epilepsy patients and healthy controls using the GEO database and various bioinformatics methods, overcoming traditional research challenges in data integration and model construction. This study has several limitations. Fifteen potential key genes in epilepsy were identified, including RPS6KA3, CTSD, and NCAM1. RPS6KA3, located on the X chromosome, encodes the RSK2 protein, crucial for neuronal growth and survival, and mutations in this gene are linked to various diseases (50). Mutations in CTSD gene, which encodes the Cathepsin D protein involved in lysosomal protein degradation, can lead to protein metabolism disorders and are associated with neurodegenerative diseases such as AD and PD (51, 52). NCAM1 gene encodes a neural cell adhesion molecule critical for neuronal interactions and network formation, and its abnormal expression is linked to various neurological disorders (53). CREG1 gene is a key transcriptional regulator, and its protein influences the cell cycle by interacting with retinoblastoma protein, impacting neuronal survival and differentiation (54). CPVL gene encodes a serine-like carboxypeptidase involved in protein degradation and immune modulation, affecting inflammatory and metabolic diseases (55). TNFRSF1A gene encodes TNFR1, a major mediator of TNF-α signaling, crucial in inflammation, apoptosis, and immune response, and is linked to various disorders, especially autoinflammatory and neurological diseases (56). PECAM1 gene encodes a transmembrane protein in endothelial cells and platelets, which is essential for angiogenesis, inflammation, and intercellular interactions, potentially influencing the blood–brain barrier and neurovascular units in epilepsy (57). IL7R gene encodes the IL-7Rα, which is crucial for immune development and regulation in T and B cells (58, 59). ZNF101 gene produces a zinc finger protein that regulates gene expression, cell cycle, and DNA repair (60). RRM2B gene encodes a subunit essential for DNA replication and repair, maintaining genome stability. MARCKSL1influences cytoskeletal dynamics and cell movement by regulating phosphatidylinositol-4,5-bisphosphate (PIP2) distribution (61). MARCKSL1 regulates cytoskeletal dynamics by controlling PIP2 distribution, aiding cell migration and invasion (62). MCM3 gene encodes a key DNA replication initiation complex component, crucial for DNA replication accuracy and genome stability (63). ATP7A gene mutations, affecting copper ion transport, are linked to copper metabolism disorders like Menkes and neurodegenerative diseases (64). MAP2K4 encodes MKK4, a dual-specificity kinase crucial in the MAPK pathway, responding to environmental stresses via JNK and p38 MAPK activation (65). Recent studies indicate that MAP2K4 is crucial in AD and PD by regulating apoptosis and neuroinflammation (66). TNC gene, which encodes an extracellular matrix protein, is abnormally expressed in neurodegenerative diseases, especially AD and PD, potentially worsening pathology by affecting cell adhesion and inflammation (67, 68). The column-line graph (Figure 6A) demonstrates that ZNF101 and MAP2K4 significantly contribute to the multifactorial logistic model. ZNF101 may influence neuronal survival by regulating DNA repair or apoptosis, while MAP2K4 is involved in the pathogenesis of epilepsy by affecting apoptosis and inflammation through the MAPK signaling pathway.

Among the 15 genes, RPS6KA3, MAP2K4, MARCKSL1, and CREG1 are associated with cell signaling, neuronal growth, and survival. Their proteins are involved in several crucial cell signaling pathways, such as MAPK and phosphatidylinositol signaling pathways. Mutations or abnormal expressions of these genes may affect neuronal excitability and plasticity, leading to abnormal neural networks and potentially causing seizures (66, 69). Besides, RRM2B, MCM3, ZNF101, CTSD, and ATP7A are involved in protein metabolism, cell cycle regulation, and genome stability, playing roles in DNA replication, repair, and protein degradation to maintain genome stability and cellular metabolic balance. Their functions are vital for neuronal survival, and any mutations or functional defects can result in disrupted protein metabolism and DNA damage accumulation, leading to neuronal damage and neurodegenerative diseases (64). NCAM1, TNC, PECAM1, IL7R, TNFRSF1A, and CPVL are associated with cell adhesion, inflammatory response, and immune regulation. The proteins encoded by these genes play essential roles in mediating intercellular interactions, facilitating the establishment of neural networks, and modulating immune system functions. Specifically, these proteins are crucial for neuronal cell adhesion, angiogenesis, and the regulation of immune responses. Abnormal expression of adhesion molecules and excessive inflammation can lead to neurodegenerative diseases (70).

Furthermore, mRNA-miRNA and mRNA-TF interaction networks were constructed to better understand the regulatory mechanisms of key genes in epilepsy. The mRNA-miRNA network revealed the role of miRNA in gene regulation, while the mRNA-TF network demonstrated the effect of TFs linked to neuronal function, stress response, and inflammation on epilepsy. These analyses addressed the limitations of gene expression data and offered new targets for personalized epilepsy treatment by regulating upstream factors of key genes. Significant regulatory interactions between 11 key genes and 72 miRNAs were identified in an mRNA-miRNA network, suggesting these miRNAs may impact epilepsy-related processes by modulating gene expression. For instance, RPS6KA3 interacts with several miRNAs (such as hsa-miR-30c-5p and hsa-miR-19a-3p), potentially affecting neuron growth and survival via the MAPK pathway, influencing neural plasticity and seizure frequency (71). Abnormal MAP2K4 expression may worsen neuronal stress and inflammation through JNK and p38 pathways, highlighting its role in epilepsy (72).

Additionally, an mRNA-TF network analysis exhibited 13 key genes significantly interacting with 40 TFs. TFs such as JUN, FOS, and STAT3 have crucial roles in cellular stress response, immune regulation, and neuronal growth by controlling key genes such as ZNF101 and TNFRSF1A. Particularly, ZNF101 influences neuronal survival and dysfunction by managing DNA repair and the cell cycle. This regulatory network is vital for understanding the gene-TF axis in epilepsy, offering insights into the pathogenesis and personalized treatment strategies for the disease. Targeting specific miRNAs or TFs can effectively manage epilepsy-related gene expression, reducing neuronal damage and seizure frequency. For instance, miR-21 influences neuronal survival via the phosphatase and tensin homolog (PTEN)/mammalian target of rapamycin (mTOR) pathway, and its intervention can mitigate epileptic seizures (72). Anti-inflammatory treatments targeting genes, including TNFRSF1A and IL7R, are essential for epilepsy patients exhibiting pronounced inflammatory responses (73, 74).

Bioinformatics and machine learning methods are used to develop an epilepsy diagnostic model using CSRGs, which lays a foundation for clinical diagnosis and personalized treatment. A multifactor logistic regression model was created using 15 key genes. The model demonstrated preliminary evidence of strong diagnostic potential based on the ROC analysis using GSE143272 and GSE32534 datasets (Figures 8B,D). This study demonstrated the effectiveness of a multifactor logistic model for diagnosing epilepsy at the genetic level, particularly using CSRG-based markers to distinguish patients from healthy individuals. This strategy combines multiple gene expressions for a more accurate prediction than single biomarker methods. The multidimensional gene combinations enhance the reliability of the model, highlighting the diagnostic potential of CSRGs. Notably, ZNF101 and MAP2K4 significantly contribute to epilepsy diagnosis (Figure 8A). ZNF101, a zinc finger transcription factor, may affect neuronal DNA repair and homeostasis. Recent research links neuronal senescence and abnormal regulatory factor expression in patients with refractory epilepsy to neuroinflammation and disease progression (9, 75). RPS6KA3, a key player in the MAPK signaling pathway, influences neuronal survival and excitability. Dysregulation of this pathway is linked to neuronal hyperexcitability and epilepsy (9, 11). MAP2K4 regulates the JNK and p38 MAPK pathways, impacting stress, apoptosis, and inflammation, and plays an important role in epilepsy-related neuronal damage and chronic inflammation (11, 76). These genes are crucial in epilepsy pathology, affecting neuroinflammation, synaptic signaling, and gene stability, offering insights into molecular mechanisms and potential therapies for epilepsy. The CSRGs-based genetic diagnostic model provides hints for personalized treatment. Early identification of high-risk epilepsy patients can help with personalized treatment strategies by detecting expression levels of key genes, including ZNF101 and MAP2K4. The role of inflammation-related genes (such as TNFRSF1A and IL7R) suggests that anti-inflammatory therapy can be a new treatment direction, especially for patients with significant inflammatory responses, potentially reducing seizure frequency.

This study also analyzed differences between high- and low-epilepsy risk groups using GSEA on 21,655 genes from the GSE4290 dataset, revealing pathways related to epileptic mechanisms (Figures 8A–E). GSEA identified significant enrichment of nervous system function pathways in the high-risk group (Figure 8B). These neurological pathways encompass neurotransmitter receptors and postsynaptic signaling mechanisms, suggesting that these genes may play a pivotal role in aberrant neuronal signaling. This dysfunction is associated with the occurrence of seizures, which are closely connected to disrupted synaptic signaling and increased neuronal excitability in epilepsy. Furthermore, the connection between epilepsy and neuroinhibitory pathways was reinforced by the enrichment of the GABA receptor signaling pathway (Figure 8C). Abnormalities in GABA receptor signaling, a key inhibitory neurotransmitter, result in reduced inhibitory neuromodulation, increasing the risk of abnormal neuronal discharges (48). These findings suggested that targeting the GABA pathway can be a potential therapeutic approach for controlling seizures. The findings also indicated that the neuroactive ligand-receptor interaction pathway (Figure 8E) was enriched in the high-risk epilepsy group. It also highlighted the enrichment of the neuroactive ligand-receptor interaction pathway in patients with high-risk epilepsy, indicating that its dysfunction may increase neuronal excitability and trigger seizures (77). Targeted intervention in this pathway can help regulate abnormal neural activity and reduce seizures. GSEA analysis linked seizures to key neurobiological pathways, particularly neurotransmitter abnormalities, GABA signaling, and neuroactive ligand-receptor interactions, offering potential targets for personalized treatments for epilepsy. Future multi-omics data research can further explore these pathways to develop new therapeutic strategies.

A total of 15 critical CSRGs were identified using bioinformatics and machine learning methodologies, and a diagnostic model was constructed that exhibited robust performance across both training and validation datasets. The primary aim of this study was to distinguish patients with epilepsy from healthy controls by leveraging gene expression profiles linked to cellular aging, thereby constructing a reliable diagnostic tool for epilepsy. The model assigned high and low scores to evaluate the likelihood of epilepsy at the time of diagnosis rather than predicting disease progression or recurrence risk. Although the validation dataset (GSE32534) was limited in size (*n* = 10), it corroborated these findings and was consistent with existing literature. Increasing the sample size can enhance the generalizability of these results. Currently, the model is in an exploratory phase and has not yet been validated at the protein level or with clinical samples. Given the constraints of time and resources, we recommend using additional external datasets or data augmentation in future research to enhance the stability and applicability of the model to a broader and more diverse population.

## 5 Conclusion

In conclusion, the findings of this study indicated a significant association between cellular senescence and the pathophysiology of epilepsy, which may present novel targets for early diagnosis and personalized treatment strategies. Considering the complexity of epilepsy as a neurological disorder, the clinical applicability of these markers warrants further investigation. Future research should focus on a comprehensive exploration of these pathways, integrating multi-omics data to substantiate their potential. This approach can yield innovative insights and strategies for the precise diagnosis and management of epilepsy. Moreover, future studies should aim to collect larger-scale epilepsy datasets through multi-center collaborations to enhance the robustness and generalizability of the model.

## Data Availability

The original contributions presented in the study are included in the article/Supplementary material, further inquiries can be directed to the corresponding author.

## Footnotes

^1^ https://www.ncbi.nlm.nih.gov/geo/

^2^ https://www.genecards.org/

^3^ https://pubmed.ncbi.nlm.nih.gov/

^4^ http://mirwalk.umm.uni-heidelberg.de/

^5^ https://rnasysu.com/chipbase3/index.php
