# Supplementary material for: Construction of epilepsy diagnosis model based on cell senescence-related genes and its potential mechanism
Source: Front Neurol. 2025 May 30;16:1555586. doi: 10.3389/fneur.2025.1555586 (PMC12162300; doi:10.3389/fneur.2025.1555586)
Supplement: Supplementary file 1 [file Data_Sheet_1.pdf]

## The link to the Raw data

Regarding the source data (raw data, initial data points) presented in the tables and figures of our manuscript, we have completed the required upload. Due to the file size exceeding 25MB, we have organized the data into a "Raw Data" folder and uploaded it to Jianguoyun. The data sharing link is as follows: https://www.jianguoyun.com/p/DcpHmI4Q7qeYDRjI8OcFIAA (Access password: lucky) .
