# Supplementary material for: Construction of epilepsy diagnosis model based on cell senescence-related genes and its potential mechanism
Source: Front Neurol. 2025 May 30;16:1555586. doi: 10.3389/fneur.2025.1555586 (PMC12162300; doi:10.3389/fneur.2025.1555586)
Supplement: Supplementary file 2 [file Data_Sheet_2.pdf]

The link to the Raw Data and Relevant Code

Regarding the source data, including raw data, initial data points, and relevant code, presented in the tables and figures of our manuscript, we confirm that the required upload has been completed. Given that the total file size exceeds 25MB, we have systematically organized these materials into a dedicated folder titled "Raw Data and Relevant Code" and uploaded it to Jianguoyun for accessibility. The data sharing link is provided as follows:

https://www.jianguoyun.com/p/DQDz20IQ9fK0DRiKg4EGIAA (Access password: RsSwHm) .
